# Commonly and Specifically Activated Defense Responses in Maize Disease Lesion Mimic Mutants Revealed by Integrated Transcriptomics and Metabolomics Analysis

**DOI:** 10.3389/fpls.2021.638792

**Published:** 2021-05-17

**Authors:** Xiaohuan Mu, Jiankun Li, Zhuangzhuang Dai, Liping Xu, Tianyuan Fan, Teng Jing, Mengyao Chen, Mingyue Gou

**Affiliations:** State Key Laboratory of Wheat and Maize Crop Science, Collaborative Innovation Center of Henan Grain Crops, College of Agronomy, Henan Agricultural University, Zhengzhou, China

**Keywords:** maize, disease lesion mimics, defense responses, transcriptomics, metabolomics, phenylpropanoid, lignin, terpenoid

## Abstract

Disease lesion mimic (*Les*/*les*) mutants display disease-like spontaneous lesions in the absence of pathogen infection, implying the constitutive activation of defense responses. However, the genetic and biochemical bases underlying the activated defense responses in those mutants remain largely unknown. Here, we performed integrated transcriptomics and metabolomics analysis on three typical maize *Les* mutants *Les4*, *Les10*, and *Les17* with large, medium, and small lesion size, respectively, thereby dissecting the activated defense responses at the transcriptional and metabolomic level. A total of 1,714, 4,887, and 1,625 differentially expressed genes (DEGs) were identified in *Les4*, *Les10*, and *Les17*, respectively. Among them, 570, 3,299, and 447 specific differentially expressed genes (SGs) were identified, implying a specific function of each *LES* gene. In addition, 480 common differentially expressed genes (CGs) and 42 common differentially accumulated metabolites (CMs) were identified in all *Les* mutants, suggesting the robust activation of shared signaling pathways. Intriguingly, substantial analysis of the CGs indicated that genes involved in the programmed cell death, defense responses, and phenylpropanoid and terpenoid biosynthesis were most commonly activated. Genes involved in photosynthetic biosynthesis, however, were generally repressed. Consistently, the dominant CMs identified were phenylpropanoids and flavonoids. In particular, lignin, the phenylpropanoid-based polymer, was significantly increased in all three mutants. These data collectively imply that transcriptional activation of defense-related gene expression; increase of phenylpropanoid, lignin, flavonoid, and terpenoid biosynthesis; and inhibition of photosynthesis are generalnatures associated with the lesion formation and constitutively activated defense responses in those mutants. Further studies on the identified SGs and CGs will shed new light on the function of each *LES* gene as well as the regulatory network of defense responses in maize.

## Introduction

Disease lesion mimics are a class of mutants that display disease-like spontaneous lesions in the absence of pathogen infection. A huge number of disease lesion mimic mutants have been found in higher plants including Arabidopsis, rice, barley, wheat, and maize (Johal et al., 1995; Lorrain et al., 2003; Rostoks et al., 2003; Gou et al., 2009; Matin et al., 2010; Tang et al., 2013). Apart from their close association with cell death in plants (Johal et al., 1995; Bruggeman et al., 2015), most of them confer enhanced resistance to diverse pathogens; disease lesion mimic mutants have therefore become keys for deciphering cell death and defense pathways in plants (Lorrain et al., 2003). In Arabidopsis, studies using over a series of lesion mimic mutants (also known as autoimmune mutants) have led to numerous breakthrough findings unraveling the complexity of plant defense responses (Lorrain et al., 2003; Van Wersch et al., 2016). For instance, *bon1* (*bonzai1*) and *cpr1/cpr30* (constitutive expressor of pathogenesis-related genes *1*/*30*) mutants that show lesion mimic phenotypes all have elevated resistance to the bacterial pathogen *Pseudomonas syringae.* Further studies on related genes indicated that they acted as negative regulators of defense responses via modulating resistance (*R*) gene *SNC1* (*Suppressor Of NPR1-1 Constitutive 1*) at transcriptional and/or protein level (Yang and Hua, 2004; Gou et al., 2009, 2012; Cheng et al., 2011; Gou and Hua, 2012). In rice, the lesion mimic mutant *oscul3a* (*Oryza sativa cullin3a*) is resistant to both *Magnaporthe oryzae* and *Xanthomonas oryzae pv oryzae*, and OsCUL3a (Cullin-3a) negatively regulates cell death and immunity by degrading OsNPR1 (Non-Expresser Of PR Genes1) (Liu et al., 2017). In barley, the *mlo* (*mildew resistance locus O*) mutant conferred race-non-specific resistance to powdery mildew pathogens (*Erysiphe graminis* f.sp. *hordei*) (Wolter et al., 1993). In wheat, the lesion mimic line Ning7840 and lm3 (*lesion mimic 3*) have enhanced resistance to leaf rust (*Puccinia triticina*) and powdery mildew (*Blumeria graminis f. sp. tritici*), respectively (Li and Bai, 2009; Wang et al., 2016).

As one of the most common classes of mutations in maize, a series of dominant disease lesion mimic (*Les*) and recessive disease lesion mimic (*les*) mutants have been found more than half a century ago (Neuffer and Calvert, 1975; Walbot et al., 1983). Although >200 *Les*/*les* loci likely exist in maize (Walbot, 1991), only several genes have been cloned so far. For instance, *lls1* (*lethal leaf spot 1*) is a *les* mutant showing elevated resistance to both *Cochliobolus heterostrophus* and *Puccinia sorghi* infections at the leaf epidermis (Simmons et al., 1998). *Rp1-D21* (*Resistance to P sorghi-D21*) mutant, a *Les* mutant resulting from an aberration in the maize *Rp1* disease resistance gene, confers a non-specific resistance to all common rust biotypes tested (*P. sorghi*) (Hu et al., 1996; Smith et al., 2010). These studies indicate that maize *Les* mutants are invaluable tools to dissect the maize defense responses conferring elevated disease resistance especially broad-spectrum resistance. However, because limited genes have been cloned, *Les/les* mutants are far from well-explored. In particular, it remains to be determined the underlying bases of the commonly activated defense responses that lead to the constitutive lesion formation, stunted growth, and enhanced disease resistance.

Transcriptional and metabolic regulations are essential for priming plant defense responses. Previous transcriptomic analysis has identified thousands of genes being upregulated under pathogen invasion (Thilmony et al., 2006; Kawahara et al., 2012; Windram et al., 2012; Dobon et al., 2016; Kebede et al., 2018), with a series of transcriptional factors being involved in controlling plant defense responses (Buscaill and Rivas, 2014). Metabolically, enhanced plant immunity largely attributes to divergent antimicrobial secondary metabolites called phytoanticipin and phytoalexin (Grayer and Harborne, 1994; Vanetten et al., 1994), for instance, the phenylpropanoids, terpenoids, benzoxazinoids, saponins, glucosinolates, etc. (Piasecka et al., 2015).

The phenylalanine-derived phenylpropanoids including lignin, flavonoids, coumarins, lignans, etc. all get involved in plant defense (Naoumkina et al., 2010). When plants suffered from pathogen infection, phenylpropanoid-related gene expressions have a significant change. For instance, *Phenylalanine Ammonia Lyase* (*PAL*) and *Cinnamyl Alcohol Dehydrogenase* (*CAD*) gene expressions are upregulated after infection of *Cercospora zeina* or *Rhizoctonia solani* in maize (Meyer et al., 2017; Li et al., 2019). Consistently, phenylalanine-derived lignin was found to be often increased after pathogen infection (Southerton and Deverall, 1990; Tiburzy and Reisener, 1990; Mohr and Cahill, 2007; Zhang et al., 2007). After treatment of lignin synthesis inhibitor, the resistance to *Puccinia graminis* was decreased in wheat (Moerschbacher et al., 1990). Recently, there have been increasing evidence supporting the idea that lignin plays vital roles in disease resistance. For instance, in maize, the lignin biosynthetic enzymes Caffeoyl-Coenzyme A O-Methyltransferase (CCoAOMT) and Hydroxycinnamoyl Coenzyme A:Shikimate Hydroxycinnamoyl Transferase (HCT) regulated plant disease resistance by forming a complex with the Nucleotide Binding Leucine-Rich Repeat (NLR) protein Rpl (Wang et al., 2015; Wang and Balint-Kurti, 2016). A further study indicated that *ZmCCoAOMT2* is involved in the resistance to multiple diseases including southern leaf blight, gray leaf spot, and northern leaf blight (Yang et al., 2017). Besides, a natural variation in the F-box gene (*ZmFBL41*) confers banded leaf and sheath blight resistance in maize, resulting from the accumulation of lignin and restriction of lesion expansion via *ZmCAD* activation (Li et al., 2019). With the discovery of new lignin biosynthetic components (Gou et al., 2018, 2019), the role of lignin in plant defense remains to be further elucidated. Other than lignin, flavonoids have been well-documented for their resistance against multiple pathogenic bacteria and fungi (Ferreyra et al., 2012; Mierziak et al., 2014). For instance, 3-deoxyanthocyanidins, one of flavonoid, accumulated in maize after *Fusarium* infection (Sekhon et al., 2006).

Other than phenylpropanoids, terpenoids and benzoxazinoids are the two most important antibiotic compound categories in maize. Terpenoids including the kauralexins class of diterpenoids and the zealexins class of sesquiterpenoids contribute largely to defense against pathogenic fungi like *Rhizopus*, *Fusarium*, *Aspergillus*, and *Colletotrichum* spp. in maize (Huffaker et al., 2011; Schmelz et al., 2011; Meyer et al., 2017). The biosynthesis of kauralexins and zealexins requires multiple genes, and their antiobiotic activities were strictly controlled (Ding et al., 2019, 2020). In addition, benzoxazinoids exhibits antibiotic activity against pathogenic fungi like *Helminthosporium turcicum*, *Cephalosporium maydis*, and *P. graminis* (Niemeyer, 2009). The biosynthesis of benzoxazinoids also consists of multiple steps via the actions of benzoxazinoneless 1 (BX1) to BX14 enzymes in maize (Handrick et al., 2016).

With the development of high-throughput transcriptomic and metabolomic techniques, the gene expression and metabolomic changes can be monitored simultaneously in order to dissect the underlying signaling and biochemical pathways in respect to specific traits (Li et al., 2010; Chen et al., 2014; Tamiru et al., 2016; Kaling et al., 2018; Mcloughlin et al., 2018; Wang et al., 2018; Castro-Moretti et al., 2020). Although *Les*/*les* mutants are considered invaluable models to dissect defense mechanisms, there is a lack of systemic characterization of maize *Les* mutants by multi-omics analysis. Therefore, in this work, we sought to determine the bases of the activated defense responses by transcriptomics and metabolomics analysis of three typical maize *Les* mutants, thereby providing new insights into the gene expression and metabolite changes associated with the lesion formation and defense-related traits in *Les* mutants.

## Materials and Methods

### Plant Material, Growth Condition, and Sampling

The *Les4* (227E Les4-N1375, maintained in W23/M14 background), *Les10* (217I Les10-NA607, maintained in W23/M14 background), and *Les17* (312B Les17-N2345, maintained in B73/A632 background) seeds were generously provided by Maize Genetics Cooperation Stock Center. All seeds were planted at the experimental station of Henan Agricultural University in Xinxiang, Henan Province, China. All *Les* mutant plants from the stock were self-pollinated for two generations. A typical line showing 1:3 segregations of wild type (WT) and mutant phenotypes in the third generation of each *Les* stock was considered to be heterozygous, and the plants with WT and mutant phenotypes were sampled separately in this generation. Specifically, the putative homozygous plants of *Les4* and *Les10* showed more intense lesions than the putative heterozygous plants, while for *Les17*, the lesion phenotype of putative homozygous and/or heterozygous plants were similar (Supplementary Figure 1). For all segregating populations, plants with uniquely intense lesion phenotype were mixed respectively to be the mutant pool, while plants with no lesion phenotype were mixed to be the WT pool. For RNA extraction and chemical analysis, the third and fourth above-ear leaves from four plants at 5 days after silking were pooled as one sample, and three replicates of sample were frozen in liquid nitrogen before use. For biomass quantification, three fully matured whole plants (without ear) per replicate were pooled as one sample and three replicates of samples were harvested and dried at 65°C to a constant weight and then weighed.

### Physiological and Biochemical Analyses

For diaminobenzidine (DAB) staining, the leaf sample harvested above were cut into 3-cm-wide pieces, soaked in 1 mg ml^–1^ DAB (*pH* = 3.8) solution for 8 h in the dark (Chintamanani et al., 2010). After removal of DAB solution, 90% ethanol was used to remove chlorophyll by incubating in a shaker. Images were taken using a stereomicroscope (Olympus SZX7). Cell wall was extracted from fresh leaves and lignin was quantified following Gou et al. (2019). Chlorophyll was measured according to Mu et al. (2016). For lignin and chlorophyll measurement, three replicates with four plants per replicate of mutant and WT were used.

### Pathogen Test of *Curvularia lunata* (Wakker) Boed.

We performed the pathogen test of *C. lunata* (Wakker) Boed. as previously described with some modifications (Huang et al., 2009). The C. lunata (Wakker) Boed. strain CX-3 was cultivated on Potato Dextrose Agar medium at 28°C for 1 week in a growth chamber. The spores were collected and suspended in distilled water with 0.02% Tween-20 and diluted into 1 × 10^6^ spores ml^–1^. The above-ear leaves of maize plants at silking stage were sprayed with the suspension and pictures taken for each leaf 7 days later. The leaves of mock treatment were sprayed with distilled water with 0.02% Tween-20. The leaf spots of the fungal colony from three representative leaves were counted using Image J software and the colony forming unit (cfu) (cm^2^ leaf area)^–1^ is presented.

### RNA Library Construction and Illumina Sequencing

Total RNA was extracted from sample using the Trizol reagent as described by Gu et al. (2013). Sequencing libraries were generated using NEBNext^®^ Ultra^TM^ RNA Library Prep Kit for Illumina^®^ (NEB, United States) following the manufacturer’s manual and index codes were added to attribute sequences to each sample (Dong et al., 2019). The average insert size for the paired-end libraries was 150 bp. Paired-end sequencing was performed on an Illumina HiSeq platform (Illumina Hiseq X-ten).

### Bioinformatics Analysis of RNA-Seq Data

Clean reads were derived after removal of low-quality regions and adapter sequences from raw reads. Then, clean reads were aligned to the maize reference genome (B73 RefGen_v4, available online: https://www.maizegdb.org/assembly/) using HISAT2 (Kim et al., 2015). Aligned reads from HISAT2 mapping were subjected to String Tie for *DeNovo* Transcript assembly (Pertea et al., 2015). The expression of each gene was normalized to fragments per kilobase of transcript per million reads (FPKM) to compare among different samples. The R package “DESeq2” was used to identify DEGs with fold changes (FC) above 2 and false discovery rate (FDR) lower than 0.05 (Love et al., 2014). The Gene Ontology (GO) and Kyoto Encyclopedia of Genes and Genomes (KEGG) analyses were accomplished in R using the packages “clusterProfiler” and “pathview” (Yu et al., 2012). The significant GO terms in the biological process were further reduced with REVIGO^1^ (Supek et al., 2011) and visualized in Cytoscape (version 3.7.1) (Shannon et al., 2003). Venn diagram was generated from the web-based Venny2.1^2^. The shared DEGs in *Les4*, *Les10*, and *Les17* were defined as common genes (CGs). The subcellular location analysis of CGs was performed with the SUBA4^3^ (Hooper et al., 2017). The heatmap was accomplished in R with the package “pheatmap.” The transcription factors (TFs) in CGs were identified based on the list obtained from PlantTFDB^4^ (Jin et al., 2017).

### Validation of RNA−Seq by Quantitative RT−PCR

Three biological replicates of the total RNA used in the RNA-seq were treated with an RNase-free DNase Kit (Cat. # RR047A, TAKARA) to remove DNA contamination (He et al., 2019) and were verified by PCR amplification using the *ZmACT1* intron primers. After being reverse transcribed into cDNA, the quantitative PCR was performed using a SYBR Green system. The primers used in the quantitative PCR analysis are listed in Supplementary Table 1. The maize *ZmACT1* gene was used as internal controls for normalizing gene expression in maize.

### Metabolomics Analysis

Metabolomics was performed at Wuhan Metware Biotechnology Co., Ltd. (Wuhan, China). The metabolites were extracted using the method of Chen et al. (2013). Briefly, using an ultrahigh-performance liquid chromatography–electrospray ionization–tandem mass spectrometry (UPLC-ESI-MS/MS) system (UPLC, Shim-pack UFLC SHIMADZU CBM30A system^5^; MS, Applied Biosystems 4500 Q TRAP^6^, the 4-μl sample extracts were injected into a C_18_ column (1.8 μm, 2.1 mm × 100 mm), which was set to 40°C. The mobile phase was used as follows: A: pure water with 0.04% acetic acid, B: acetonitrile with 0.04% acetic acid. Sample measurements were performed with a gradient program as follows: 0–10 min, 5% B–95% B; 10–11 min, 95% B; 11–11.1 min, 95% B–5% B; 11.1–14 min, 5% B. The effluent was alternatively connected to an ESI-triple quadrupole-linear ion trap (QTRAP)-MS. The conditions and operation parameters were set as in previous studies (Zeng et al., 2020). Metabolite quantification was performed using multiple-reaction monitoring (MRM) mode (Shi et al., 2020). Partial least squares discriminant analysis (PLS-DA) was used to study the identified metabolites. Differentially accumulated metabolites (DAMs) were set with thresholds of variable importance in projection (VIP) ≥ 1 and log_2_(FC) ≥ 1 or ≤ -1. The shared DAMs in *Les4*, *Les10*, and *Les17* were defined as common differentially accumulated metabolites (CMs). KEGG analysis of CMs was performed with the MBROLE 2.0^7^ (Lopezibanez et al., 2016).

## Results

### Phenotypic and Physiological Characterization of the *Les4*, *Les10*, and *Les17* Mutants

**FIGURE 1:**
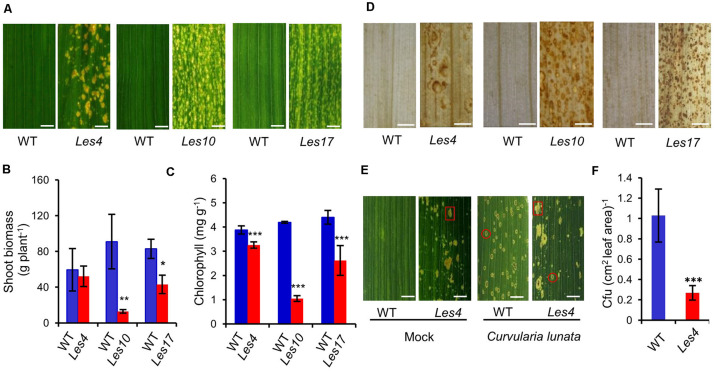
Phenotypic and physiological characterization of the *Les4*, *Les10*, and *Les17*. **(A)** Morphologies of *Les4*, *Les10*, and *Les17* mutants and their respective wild type (WT). Scale bars = 5 mm. **(B)** The shoot biomass and content of chlorophyll in *Les4*, *Les10*, and *Les17* mutants and their respective WT. **(C)** The total chlorophyll content in *Les4*, *Les10*, and *Les17* mutants and their respective WT. **(D)** Images of DAB-stained leaves of in *Les4*, *Les10*, and *Les17* mutants and their respective WT. Scale bars = 2 mm. **(E)** Morphologies of Mock and *Curvularia lunata*-infected WT and *Les4* plant leaves 7 days after inoculation. A typical spontaneous lesion was indicated by a red square, and a typical *curvularia*-leaf spot-disease lesion was indicated by a red circle. Scale bars = 7.5 mm. **(F)** Quantification of *Curvularia lunata* colonies in Mock and *Curvularia lunata*-infected WT and *Les4* plant leaves 7 days after inoculation. For **(B,C,F)**, asterisks indicate significant differences compared with WT samples (Student’s *t*-test; *P < 0.05; **P < 0.01; ***P < 0.001). Error bars represent standard deviation.

To explore the molecular bases of lesion formation and defense-related traits in *Les* mutants, we used three representative *Les* mutants *Les4*, *Les10*, and *Les17* that have been previously mapped to distinct loci (Johal et al., 1995). Because the three mutants are maintained as heterozygotes in different background and the phenotype of *Les* mutants can be affected largely by genetic background according to previous report (Hoisington et al., 1982), all *Les* mutants from the original stock were self-pollinated for two generations, and the segregation population of the third generation was examined phenotypically and physiologically. Compared with their relative WT, *Les4*, *Les10*, and *Les17* all showed spontaneous necrotic lesions on the leaves (Figure 1A and Supplementary Figure 1). In particular, *Les4* mutant developed large necrotic lesion at later growth stage, *Les10* displayed medium lesion at early stage, and *Les17* showed small lesion at medium stage, consistent with a previous report (Johal et al., 1995). The shoot biomass was 86 and 48% lower than WT in *Les10* and *Les17*, respectively (Figure 1B), while that of *Les4* was only slightly but non-significantly lower than WT. Because all mutants showed yellowish phenotype, we measured their total chlorophyll content. Compared with their relative WT plants, all *Les* mutants have significantly reduced chlorophyll content (Figure 1C). Consistent with the necrotic lesions being observed, increased accumulation of H_2_O_2_ could be visualized in all three mutants by DAB staining following a previously described method (Chintamanani et al., 2010; Figure 1D).

We tested the disease resistance of *Les4* using a *C. lunata* (Wakker) Boed. strain that causes *curvularia* leaf spot because the oval-shaped disease lesions are easily distinguishable from that of the big irregularly shaped spontaneous lesions in *Les4*. At 7 days after inoculation, the WT leaves displayed many disease lesions, while disease lesions observed in *Les4* mutant were about 25% that of the WT, indicating significantly enhanced resistance of *Les4* to *curvularia* leaf spot (Figures 1E,F).

### Identification of the Differentially Expressed Genes Between WT and Mutant

We carried out transcriptomic analysis of *Les* mutants and their respective WT by RNA sequencing based on Illumina HiSeq platform. We used the third and fourth above-ear leaves at 5 days after silking because the lesion became easily visible at this stage, and the leaves were still in highly vigorous state. To eliminate the effect of background, the leaves of four plants were pooled as one sample, and three replicates of sample were used for RNA extraction and sequencing. After sequencing, 32,025, 33,031, and 32,035 expressed genes were detected in *Les4*, *Les10*, and *Les17*, respectively. The principal components analysis (PCA) plots clearly separated the WT samples from the mutant samples and the replicates of both WT and mutants were clustered into distinct patches (Supplementary Figure 2A), suggesting good reliability of our RNA-seq data.

**FIGURE 2:**
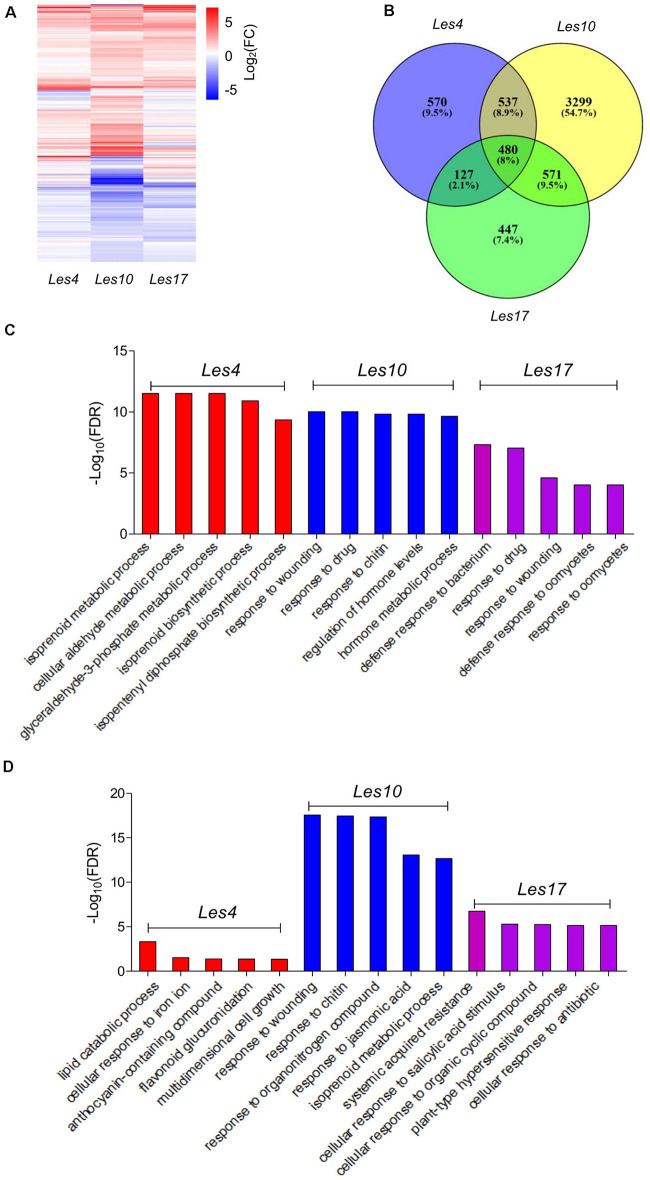
Transcriptomic analysis of *Les4*, *Les10*, and *Le17*. **(A)** Heatmap showing the differentially expressed genes (DEGs) in *Les4*, *Les10*, and *Les17.* **(B)** Venn diagram displaying DEGs unique and common in mutant vs. wild type in *Les4*, *Les10*, and *Les17.* **(C)** The top 5 GO terms of GO analysis of all DEGs in *Les4*, *Les10*, and *Les17.* **(D)** The top 5 GO terms of GO analysis of specific DEGs in *Les4*, *Les10*, and *Les17*.

A total of 1,714, 4,887, and 1,625 differentially expressed genes (DEGs) were identified in *Les4*, *Les10*, and *Les17*, compared to their respective WT, respectively (Figure 2A and Supplementary Tables 2, 3). Of these genes, 1,334, 2,861, and 1,134 were upregulated while 380, 2,026, and 491 were downregulated. More DEGs were identified in *Les10* than in *Les4* and *Les17* (Supplementary Table 3). Furthermore, well-matched qRT-PCR results to the expression data of RNA-seq indicated reliability of our RNA-seq analysis (Supplementary Table 4). GO term enrichment analysis was performed to elucidate the functional enrichment of DEGs in each mutant. There were 187 biological processes (BPs), 17 cellular components (CCs), and 2 molecular functions (MFs) in GO analysis of DEGs of *Les4*. DEGs of *Les4* were mainly related to “isoprenoid metabolic process,” “cellular aldehyde metabolic process,” “glyceraldehyde-3-phosphate metabolic process,” “isoprenoid biosynthetic process,” and “isopentenyl diphosphate biosynthetic process” (Figure 2C and Supplementary Table 5) in BP-GO terms. There were 270 BPs, 11 CCs, and 63 MFs in GO analysis of DEGs of *Les10*. DEGs of *Les10* were mainly related to “response to wounding,” “response to drug,” “response to chitin,” “response to hormone levels,” and “hormone metabolic process” in BP-GO terms (Figure 2C and Supplementary Table 5). There were 129 BPs and 52 MFs in GO analysis of DEGs of *Les17*. DEGs of *Les17* were mainly related to “defense response to bacterium,” “response to drug,” “response to wounding,” “defense response to oomycetes,” and “response to oomycetes” in BP-GO terms (Figure 2C and Supplementary Table 5). In general, the DEGs were mostly related to defense response and metabolic process.

### Identification of the Specific Differentially Expressed Genes in Different *Les* Mutants

A total of 570, 3,299, and 447 DEGs were specifically expressed in *Les4*, *Les10*, and *Les17* mutants and were defined as specific genes (SGs) (Figure 2B). To look into the specificity of each *Les* mutant, GO enrichment analysis was also performed for SGs in each mutant. SGs of *Les4* were mainly related to “lipid catabolic process,” “cellular response to iron ion,” “anthocyanin-containing compound,” and “flavonoid glucuronidation” (Figure 2D and Supplementary Table 5). SGs of *Les10* were mainly related to “response to wounding,” “response to chitin,” “response to organonitrogen compound,” and “response to jasmonic acid” (Figure 2D and Supplementary Table 5). SGs of *Les17*, however, were mainly related to “systemic acquired resistance,” “cellular response to salicylic acid stimulus,” “cellular response to organic cyclic compound,” and “plant-type hypersensitive response” (Figure 2D and Supplementary Table 5). We searched for all SGs in the Pathogen Receptor Genes (PRGs) database^8^, a database of plant resistance genes, and found 31, 167, and 58 genes for *Les4*, *Les10*, and *Les17*, respectively (Supplementary Table 6), suggesting that those PRGs were specifically regulated in different *Les* mutants.

### Identification of the Common Differentially Expressed Genes in Different *Les* Mutants

**FIGURE 3:**
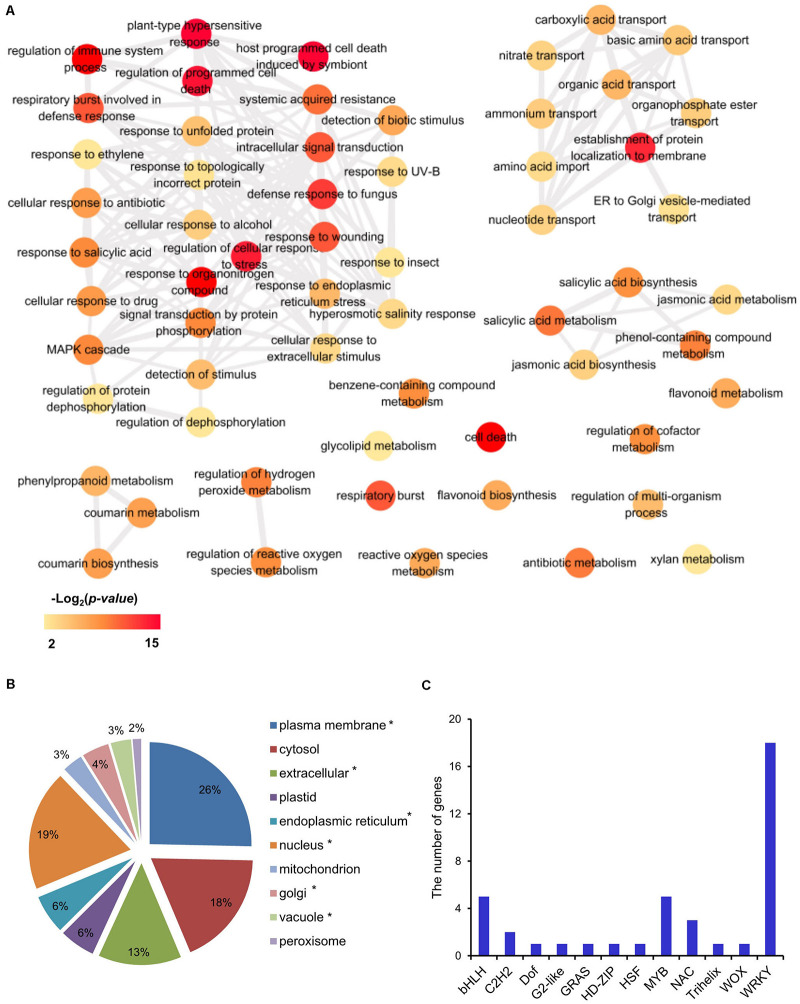
Transcriptomic analysis of common differentially expressed genes (CGs) in mutant vs. wild type in *Les4*, *Les10*, and *Les17.* **(A)** Biological process of gene ontology (GO) term analysis of CGs. The GO term analysis was conducted in R with packages “clusterProfiler.” The full list of significant GO terms (Supplementary Table 5) assigned into biological process were subjected into REViGO (http://revigo.irb.hr/) to redundant GO terms, and visualized in Cytoscape. The color indicated the significance [−Log_2_(*p*-value)] of GO terms. Only significant GO terms were shown. **(B)** The proportion of cellular location of proteins encoded by common genes (CGs) in *Les4*, *Les10*, and *Les17*. The asterisk behind each item indicates that the *p*-value of the hypergeometric distribution test was lower than 0.05. **(C)** The gene numbers of each transcription factor family in CGs.

Since the three *Les* mutants are in different backgrounds, it is less likely that we could identify many common DEGs. However, 480 DEGs were commonly shared by the *Les4*, *Les10*, and *Les17* mutants and were designated as CGs (Figure 2B). GO enrichment analysis was also performed for CGs. There were 114 BPs and 13 MFs in GO analysis of CGs (Supplementary Table 5). The 114 BP-GO terms were subjected to REVIGO software based on their relationship and then reassigned to 60 terms (Figure 3A). The top GO terms with high significance include “regulation of programmed cell death,” “regulation of immune system process,” “plant-type hypersensitive response,” “respiratory burst involved in defense response,” and “host programmed cell death induced by symbiont.” Interestingly, all these GO terms are related to plant defense responses. Many previously published defense-related genes in maize were upregulated in the *Les* mutants (Table 1) including the *NLR* genes, the receptor like kinase genes, the zealexins and kauralexins biosynthetic genes, and the pathogenesis-related (*PR*) genes. We specifically searched for the 480 CGs in the PRGs database and identified 58 putative PRGs upregulated in all three *Les* mutants (Supplementary Table 7). In addition, through a comparison of the 480 CG genes to the published transcriptomics data using Plant Regulomics online software^9^ (Ran et al., 2020), up to 384 out of 475 (∼81%) listed genes are shared with the published genes being induced by pathogen infection (Supplementary Table 8). Those data further indicate that the expression of defense-related genes was generally induced in all *Les* mutants, and substantial characterization of those genes may lead to interesting discovery dissecting the general nature of the lesion formation and constitutively activated defense responses in those mutants.

**TABLE 1:** Representative common differentially expressed genes involved in plant defense response.

ID	Log2FC				
	Les4	Les10	Les17	NR_annotation/Gene symbol	References
Zm00001d052992	1.37	1.48	2.05	Disease resistance protein RPM1	Cheng et al., 2012
Zm00001d014649	1.55	2.67	2.68	Disease resistance protein RPM1	Cheng et al., 2012; Song et al., 2015
Zm00001d021492	1.91	1.31	3.25	Putative disease resistance protein RGA3	Cheng et al., 2012
Zm00001d053424	2.65	1.52	2.86	Receptor-like protein kinase 5	Song et al., 2015
Zm00001d024210	1.23	2.32	3.15	TPS11/ZX3	Ding et al., 2020
Zm00001d024208	2.19	7.59	8.19	TPS12/Zx2	Ding et al., 2020
Zm00001d024211	4.36	6.73	6.45	TPS13/Zx4	Ding et al., 2020
Zm00001d014121	2.55	4.40	4.76	ZX5/CYP71Z19	Ding et al., 2020
Zm00001d034097	3.17	5.11	1.43	CYP81A39/ZX10	Ding et al., 2020
Zm00001d014134	1.46	3.83	3.14	ZX6/CYP71Z18	Ding et al., 2020
Zm00001d041082	2.00	3.91	3.55	KSL2	Ding et al., 2019
Zm00001d046342	1.62	3.87	2.28	KO2	Ding et al., 2019
Zm00001d032858	1.24	3.51	−1.25	KSL4	Ding et al., 2019
Zm00001d029648	1.71	3.43	2.63	CPPS2/AN2	Christensen et al., 2018; Mafu et al., 2018
Zm00001d034365	1.29	3.25	1.55	PEN1	Wu et al., 2015
Zm00001d003023	2.13	1.56	2.15	RFO1	Wu et al., 2015
Zm00001d043238	1.96	1.84	2.54	PR9	Wu et al., 2015
Zm00001d017152	1.48	1.99	2.67	PR4	Wu et al., 2015
Zm00001d048021	1.22	4.53	2.96	AOS	Zuo et al., 2015
Zm00001d003379	4.26	−2.06	−4.81	Pathogenesis−related protein	Yu et al., 2018
Zm00001d034460	3.03	−6.54	−1.70	IGL1	Ahmad et al., 2011; Zhou et al., 2018
Zm00001d029359	2.04	5.28	1.70	BX10	Zhou et al., 2018
Zm00001d007718	4.64	−2.10	1.52	BX13	Zhou et al., 2018
Zm00001d004921	2.45	3.80	1.74	BX14	Zhou et al., 2018

In the GO category of metabolism of CGs, flavonoid, salicylic acid (SA), jasmonic acid (JA), and phenylpropanoid were significantly enriched (Figure 3A). Consistently, KEGG analysis also indicates that CGs were mainly enriched in pathways related to phenylpropanoid and flavonoid biosynthesis (Supplementary Table 9), and thiamine and diterpenoid biosynthesis were also enriched (Supplementary Table 9). In addition, almost all genes involved in regulating reactive oxygen species (ROS) production were upregulated in all *Les* mutants vs. their relative WT (Table 2), which is consistent with the increased H_2_O_2_ accumulation as shown by DAB staining in those *Les* mutants (Figure 1C).

**TABLE 2:** Common differentially expressed genes involved in generation of reactive oxygen species.

ID	Log_2_(FC)			Annotation
	*Les4*	*Les10*	*Les17*	
Zm00001d016182	−1.24	−1.40	−1.83	Peroxidase 52
Zm00001d042022	1.16	3.61	1.92	Peroxidase 12
Zm00001d037550	1.43	2.15	2.44	Peroxidase 5
Zm00001d007301	1.68	2.14	1.76	Protein disulfide isomerase 2
Zm00001d040705	1.87	4.25	2.86	Peroxidase 64
Zm00001d043238	1.96	1.84	2.54	Peroxidase 56
Zm00001d037547	2.01	5.47	4.17	Peroxidase 5
Zm00001d002901	2.09	1.46	3.52	Peroxidase 12 precursor
Zm00001d002899	3.15	2.45	3.12	Peroxidase 12

Based on SUBA4 analysis, most proteins encoded by those CGs were estimated to localize in plasma membrane and nucleus (Figure 3B). Consistent with their dominant nucleus localization, 40 TFs were found, as predicted by PlantTFDB software (Jin et al., 2017). These TFs belong to 12 gene families, among which, WRKY, MYB, and bHLH rank the top three most enriched TF families (Figure 3C and Supplementary Table 10).

### Identification of Differentially Accumulated Metabolites Between WT and Mutant

**FIGURE 4:**
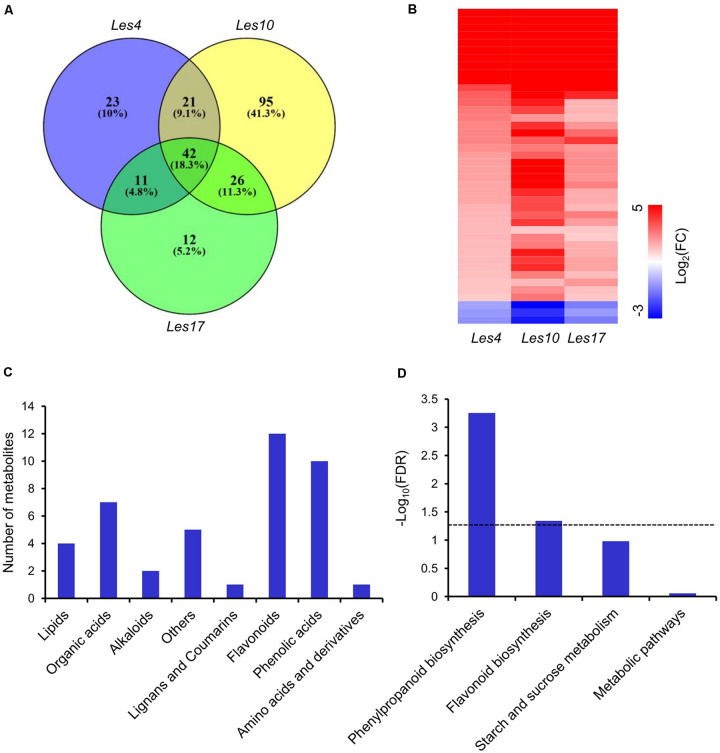
Metabolomics analysis of *Les4*, *Les10*, and *le17*. **(A)** Venn diagram displaying differentially accumulated metabolites unique and common in mutant vs. wild type in *Les4*, *Les10*, and *Les17*. **(B)** Heatmap showing the differentially accumulated common metabolites in *Les4*, *Les10*, and *Les17.* **(C)** The number of common differentially accumulated metabolites (CMs) in different metabolite categories. **(D)** Kyoto Encyclopedia of Genes and Genomes (KEGG) analysis of CMs. The dash line represents a cutoff of *p* = 0.05.

We carried out widely targeted metabolomics assay of *Les* mutants and their relative WT by UPLC-ESI-MS/MS. In this assay, 455 metabolites were collectively identified, including ∼30% flavonoids, 13% phenolic acids, 12% lipid, 11% alkaloids, 11% amino acids and derivatives, 6% organic acids, 5% nucleotides and derivatives, 2% lignans and coumarins, and 10% others (Supplementary Figure 3 and Supplementary Table 11). PCA showed that all biological replicates for each group were clustered closely (Supplementary Figure 2B), suggesting high reproducibility and reliability. We found 97, 184, and 91 differentially accumulated metabolites (DAMs) in *Les4*, *Les10*, and *Les17*, respectively. Of these metabolites, 94, 134, and 66 were upregulated while 3, 50, and 25 were downregulated in *Les4*, *Les10*, and *Les17*, respectively (Supplementary Tables 2, 11). Venn diagram indicates that 42 DAMs were commonly shared by all three *Les* mutants and were defined as common metabolites (CMs) (Figure 4A and Supplementary Table 12). In those CMs, only 3 DAMs were downregulated while 39 were upregulated (Figure 4B and Supplementary Table 12). Interestingly, the fold change of metabolites in *Les10* was larger than in *Les4* and *Les17*. This is consistent with the larger amount of DEGs in *Les10* (Figure 2A). Among all CMs, flavonoids, phenolic acids, and organic acids ranked the top 3 most represented metabolites (Figure 4C). While for KEEG pathway enrichment analysis, pathways including phenylpropanoid biosynthesis and flavonoid biosynthesis were significantly enriched (Figure 4D).

### Integrated Transcriptomics and Metabolomics Analysis of Lignin and Flavonoid Biosynthesis

Based on KEGG and GO enrichment analysis of CGs and CMs, we found that terms of phenylpropanoids and flavonoids were most highly enriched (Figure 4D and Supplementary Tables 5, 9). Therefore, an integrated transcriptomics and metabolomics analysis specifically on phenylpropanoid, lignin, and flavonoid biosynthesis was applied in this study.

**FIGURE 5:**
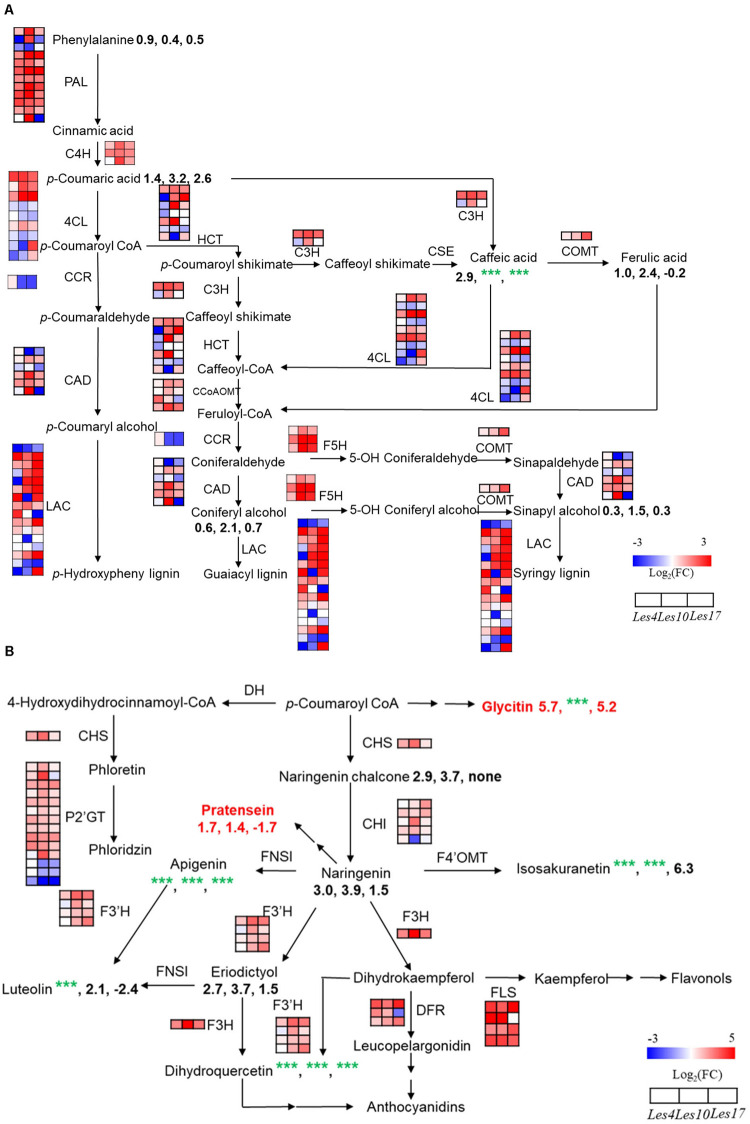
Integrated transcriptomics and metabolomics analysis of lignin and flavonoid biosynthesis. **(A)** Genes and metabolites involved in lignin biosynthetic pathway in the comparisons of *Le4*, *Les10*, and *Les17* mutants and their respective wild type (WT). **(B)** Genes and metabolites involved in flavonoid biosynthetic pathway in the comparisons of *Le4*, *Les10*, and *Les17* mutants and their respective WT. For **(A,B)**, the different rows of each heatmap represent the different homolog genes for each enzyme, with each column representing different genotypes and different colors indicating different gene expression levels. The number below each metabolite is the log_2_ (fold change) value of it. Red text highlights the compounds belonging to isoflavone. The detailed information of genes and metabolites involved in lignin and flavonoid biosynthesis are available in Supplementary Tables 13–16, respectively. Double arrows in sequence represent omitted metabolites and genes. nd, not detected. CAD, Cinnamyl Alcohol Dehydrogenase; CCR, Cinnamoyl-Coenzyme A Reductase; C3H, Cinnamate 3-Hydroxylase; C4H, Cinnamate 4-Hydroxylase; CHS, Chalcone Synthase; CHI, Chalcone Isomerase; 4CL, 4-Coumarate:Coenzyme A Ligase; CCoAOMT, Caffeoyl-Coenzyme A O-Methyltransferase; COMT, Caffeic Acid O-Methyltransferase; CSE, Caffeoyl Shikimate Esterase; DFR, Dihydroflavonol 4-Reductase; DH, Dehydrogenase; FLS, Flavonol Synthase; FNS, Flavone Synthase; F3H, Flavanone 3-Hydroxylase; F3’H, Flavanoid 3’-Hydroxylase; F4’OMT, Flavonoid 4’-O-Methyltransferase; F5H, Ferulate 5-Hydroxylase; HCT, Hydroxycinnamoyl Coenzyme A:Shikimate Hydroxycinnamoyl Transferase; LAC, Laccase; PAL, Phenylalanine Ammonia Lyase; P2’GT, UDP-Glucose:Phloretin 2’-O-Glucosyltransferase.

**FIGURE 6:**
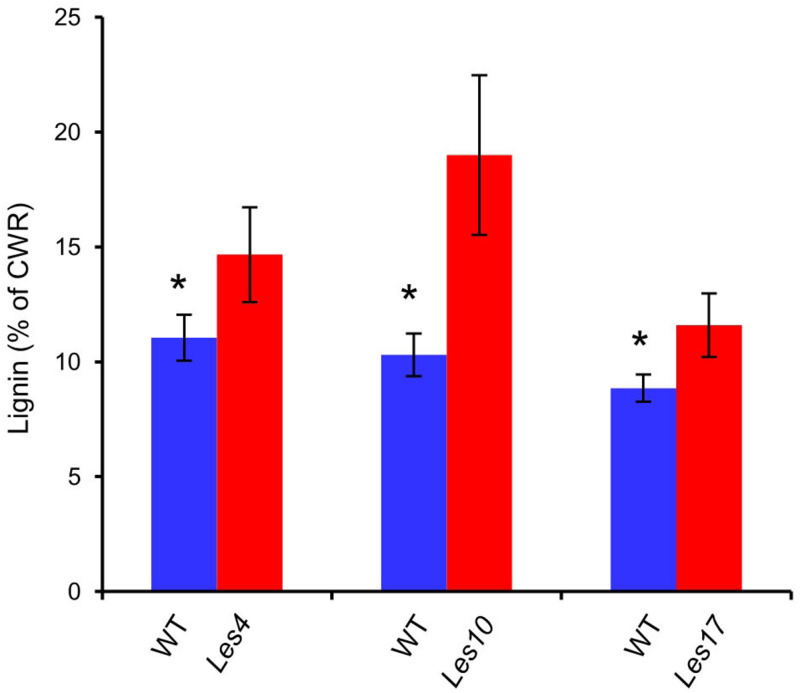
Total lignin content in *Les4*, *Les10*, and *Les17* mutants and their respective wild type. CWR, cell wall residues. Asterisks indicate significant differences compared with wild type (WT) samples (Student’s *t*-test; *P < 0.05). Error bars represent standard deviation.

Most of the genes encoding the key enzymes in phenylpropanoid and lignin biosynthesis, including *PAL*, Cinnamate 4-Hydroxylase (*C4H)*, 4-4-Coumarate: Coenzyme A Ligase (*4CL*), *HCT*, *CAD*, Laccase (*LAC*), etc., displayed increased expression in *Les4*, *Les10*, and *Les17* mutant compared to their respective WT (Figure 5A and Supplementary Table 13). Most of the phenylpropanoid compounds, e.g., *p*-coumaric acid, caffeic acid, and coniferyl alcohol, were increased in all *Les* mutants compared with WT (Figure 5A and Supplementary Table 14). *p*-coumaric acid, for instance, was increased 2.72, 9.09, and 5.89 times in *Les4*, *Les10*, and *Les17* mutants, respectively. Besides, we performed acetyl bromide lignin measurement of all *Les* mutants and their respective WT. The lignin content was 33, 85, and 31% higher in mutant vs. WT in *Les4*, *Les10*, and *Les17*, respectively (Figure 6). Taken together, these data indicate that all three *Les* mutants have altered phenylpropanoid biosynthetic gene expression as well as higher phenylpropanoid and lignin accumulation.

Most flavonoid biosynthetic genes, including *Chalcone Synthase* (*CHS*), *Chalcone Isomerase* (*CHI*), *Flavonol Synthase* (*FLS*), UDP-*Glucose:Phloretin 2’-O-Glucosyltransferase* (*P2’GT*), *Flavanone 3-Hydroxylase* (*F3H*), *Flavonoid 3’-Hydroxylase* (*F3’H*), and *Dihydroflavonol 4-Reductase* (*DFR*), were greatly increased in *Les* mutants (Figure 5B and Supplementary Table 15). The levels of associated metabolites including dihydroquercetin, naringenin, apigenin, eriodictyol, luteolin, etc. were increased dramatically in the *Les* mutants (Figure 5B and Supplementary Table 16). For example, eriodictyol was increased 6. 30-, 13. 05-, and 2.84-fold in *Les4*, *Les10*, and *Les17* mutants compared with their respective WT (Supplementary Table 16), and this is consistent with the substantial rises in the *F3H* expression level. Besides, the isoflavone compounds, glycitin, and pratensein, also accumulated significantly in *Les4* and *Les10* mutants (Figure 5B and Supplementary Table 11).

### Terpenoid and Benzoxazinoid Biosynthesis in *Les* Mutants

**FIGURE 7:**
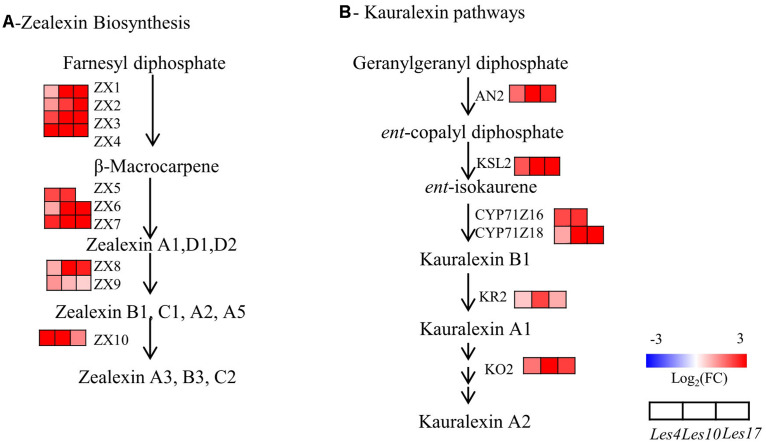
The transcriptomics analysis of zealexins and kauralexins biosynthesis. **(A)** Transcriptional changes of zealexins biosynthetic genes in *Le4*, *Les10*, and *Les17* mutants compared to their respective wild type (WT). **(B)** Transcriptional changes of kauralexins biosynthetic genes in *Le4*, *Les10*, and *Les17* mutants compared to their respective WT. For **(A,B)**, different rows represent the different homolog genes encoding the related enzyme, with each column representing different genotypes and different colors indicating different gene expression levels. The number below each metabolite is the log_2_ (fold change) value. The detailed information of genes involved in zealexins and kauralexins biosynthesis is available in Supplementary Table 17. Double arrows in sequence represent omitted metabolites and genes. ZX, Zealexin; TPS, Terpene Synthase; AN2, Anther Ear 2; KSL, Kaurene Synthase-Like, CYP, Cytochrome P450, KO2, Kaurene Oxidase 2; KR2, Kauralexin Reductase 2.

We specifically looked into the biosynthesis of terpenoids (zealexins and kauralexins) and benzoxazinoid since both are very important antibiotic compounds in maize. Because zealexins and kauralexins were not detected due to technical issue in this study, we specifically checked the transcriptional changes of zealexins and kauralexins biosynthetic genes. Surprisingly, all of the zealexins biosynthetic genes, *Zealexin* (*ZX*) *1-10* (*ZX1*-*ZX10*), were upregulated in all *Les* mutants (Figure 7 and Supplementary Table 17). Similarly, all kauralexins biosynthetic genes including *Anther Ear* 2 (*AN2*), *Kaurene Synthase-Like* (*KSL2*), *Cytochrome P450 (CYP) Z16/18* (*CYP71Z16*/*18*), *Kauralexin Reductase 2* (*KR2*), and *Kauralexin Oxidase 2* (*KO2*) were also upregulated in *Les* mutants (Figure 7 and Supplementary Table 17). These data collectively indicate that biosynthesis of zealexins and kauralexins are generally activated in *Les* mutants. In addition, according to the metabolomics data, there was a common decrease of DIMBOA-Glucoside (Supplementary Figure 4 and Supplementary Table 11), the dominant benzoxazinoid compound with strong antifungal activity, in all three *Les* mutants. Interestingly, the upstream benzoxazinoid biosynthetic genes *Benzoxazinless* (BX) 1–6 (*BX1*–*BX6*) were mostly downregulated in almost all three *Les* mutants (Supplementary Figure 4 and Supplementary Table 18). In contrast, the downstream benzoxazinoid biosynthetic genes *BX10*, *BX11*, *BX12*, and *BX14* genes were mostly upregulated in those *Les* mutants (Supplementary Figure 4 and Supplementary Table 18). These data indicate that while the biosynthesis of DIMBOA-Glucoside was repressed, the processing of DIMBOA-Glucoside into HDMBOA-Glucoside was generally activated in *Les* mutants.

## Discussion

Plant *Les/les* mutants show spontaneous lesion and enhanced disease resistance that are often accompanied by stunted plant growth, implying the constitutive activation of defense responses in those mutants. To make better use of the maize *Les/les* mutants, it is crucial to systematically understand the molecular bases of commonly and specifically activated defenses leading to those traits. In this study, high-throughput transcriptomics and metabolomics analysis were utilized to explore the specific and common transcriptional and metabolic changes associated with the *Les*-related traits including necrotic lesions, stunted plant growth, and enhanced disease resistance.

In this study, we identified 1,714, 4,887, and 1,625 DEGs in *Les4*, *Les10*, and *Les17*, respectively. According to GO enrichment analysis, most of those DEGs were related to defense response and metabolic process (Figure 2C and Supplementary Table 5). To dissect the specificity of each *Les* mutant and the potential function of each *LES* gene, we looked into those SGs in each mutant (570, 3,299, and 447 are SGs for *Les4*, *Les10*, and *Les17*, respectively). GO enrichment analysis of SGs indicate that *LES4*’s function may be more directly related to secondary metabolism regulation since lipid, anthocyanin, and flavonoid metabolic processes were most significantly changed in *Les4*. *LES10* is probably involved in the regulation of defense responses to necrotrophic pathogen since wounding and JA-related processes were most significantly changed. *LES17*, however, is likely involved in the regulation of SA-mediated systemic acquired resistance and hypersensitive responses since representative genes were captured. Using PRG database, we identified 31, 167, and 58 PRGs for *Les4*, *Les10*, and *Les17*, respectively (Supplementary Table 6), which also support the specific regulation of defense responses in different *Les* mutants. Further studies on those SGs will provide more clues to the biological function of each *LES* gene.

We found that the total DEGs and DAMs in *Les10* are generally more abundant than *Les4* and *Les17*, likely due to more intense lesion observed in *Les10* since its lesion formed at earlier stage. While designing the experiments, two aspects may affect the results in general. Firstly, different timing of sampling will surely affect the intensity of lesion and thus the gene expression change and metabolite accumulation. We sampled the third and fourth above-ear leaves at 5 days after silking, although the leaves of all mutants are still in good vigor, the relatively “matured” state may lead to substantial changes of gene expression and metabolite levels. Secondly, because of the non-uniform nature of the genetic backgrounds of the three mutants, there is lack of common reference for changes in gene expression levels and metabolite levels. Both aspects argue against the joint analysis of the three mutants; however, we did identify a significantly large amount of CGs (480) for all three *Les* mutants. More interestingly, ∼81% of CGs are shared with the published genes being induced by pathogen infection (Supplementary Table 8), representing robust changes of general defense-related gene expression. Although it is possible that those robust changes of general defense-related gene expression are largely due to general responses accompanying the lesion formation, those changes allow us to digest the shared regulatory network controlling the generally activated defense responses in *Les* mutants. Based on further in-depth analysis, we found that the transcriptional and metabolic changes are closely associated with each common trait of the *Les* mutants, which are organized and discussed below.

### Transcriptional and Physiological Changes Associated With Necrotic Lesion Formation and Stunted Plant Growth

Necrotic lesion is caused by programmed cell death, a process including cytoplasmic shrinkage, chromatin condensation, mitochondrial swelling, vacuolization, and chloroplast disruption (Coll et al., 2011). Indeed, our transcriptomic analysis indicates that many DEGs in *Les* mutants are cell death related. Specially, genes involved in ROS accumulation including several peroxidase coding genes are mostly upregulated in all tested *Les* mutants (Table 2). Consistently, all tested *Les* mutants have higher H_2_O_2_ accumulation and lower chlorophyll content (Figures 1C,D). Previous studies indicate that chlorophyll perturbation can also lead to ROS production and consequently plant cell death. For instance, *LES22* encodes a key enzyme in the biosynthetic pathway of chlorophyll, and accumulation of photo-excitable uroporphyrin leads to minute necrotic spots in *Les22* (Hu et al., 1998). Similarly, the light-dependent cell death of the *les* mutant *lls1* is caused by failure of chlorophyll breakdown (Gray et al., 1997, 2004). The reduced shoot biomass may be partly due to the lower photosynthesis accompanied by lower chlorophyll content since chlorophyll plays a vital role in photosynthesis (Mu et al., 2016). We found 57 differentially regulated photosynthesis-related genes, including light reaction, Calvin cycle, and carbon concentration, while 3, 42, and 7 of them were downregulated in *Les4*, *Les10*, and *Les17*, respectively (Supplementary Table 19). These data support the tight correlation between lower photosynthesis and compromised plant growth since *Les4*, *Les10*, and *Les17* show mild, intense, and medium reduction of biomass, respectively. In summary, the upregulation of cell death-related genes and the accumulation of ROS is associated with the necrotic lesion formation while the lower photosynthesis rate likely contributes to the reduced plant growth in *Les* mutants.

### Transcriptional Changes Associated With Enhanced Disease Resistance in *Les* Mutants

In this study, we found that *Les4*, *Les10*, and *Les 17* mutants showed constitutively activated defense responses. For instance, *Les4* was highly resistant to *curvularia* leaf spot (Figures 1E,F). Consistently, GO enrichment analysis of CGs indicates that most terms were related to plant defense (Supplementary Table 5). Besides, most CGs are shared with genes differentially expressed after pathogen infection according to the Plant Regulomics database (Supplementary Table 8), and PRGs were enriched in both CGs and SGs (Supplementary Tables 6, 7). Although relatively less defense-related genes have been verified in maize, we did find a few previous reported maize defense genes in CGs, including *NLR* genes, the receptor-like kinase genes, the zealextins and kauralexins biosynthetic genes, and *PR* genes (Table 1).

Of course, we should always be cautious about the identified DEGs considering that many genes could be consequences of general metabolic changes due to the lesion formation of *Les* mutants. We therefore tried to look at the whole regulatory network by checking TFs that likely control downstream defense gene expression and metabolic changes. Among the 480 CGs, there are 40 TFs mainly belonging to WRKY, bHLH, and MYB families (Figure 3C and Supplementary Table 10). WRKY and MYB TFs have previously been found to play broad and pivotal roles in regulating plant disease resistance (Eulgem and Somssich, 2007; Pandey and Somssich, 2009; Liu et al., 2013). In our research, 18 WRKY and 5 MYB TFs were upregulated in all three *Les* mutants (Supplementary Table 10), implying their contributions to the enhanced resistance. Interestingly, *WRKY79* (*Zm00001d020137*), which was previously shown to positively regulate zealexins and kauralexins biosynthesis (Fu et al., 2018), was upregulated in all *Les* mutants (Supplementary Table 10), consistent with the upregulation of the terpenoid biosynthetic genes (Figure 7 and Supplementary Table 17). Previous studies also indicated that a series of MYB TFs were involved in the transcriptional regulation of lignin and flavonoid biosynthesis (Zhao and Dixon, 2011; Geng et al., 2020; Wang et al., 2020); some of the MYB TFs identified in this study may be potentially involved in lignin and flavonoid biosynthesis in maize. Taken together, we propose that the upregulation of defense-related genes and secondary metabolite biosynthetic genes potentially mediated by WRKY and MYB TFs is closely associated with the enhanced disease resistance in *Les* mutants.

### Metabolic Changes Associated With Enhanced Disease Resistance in *Les* Mutants

Genes involved in secondary metabolite biosynthesis are also highly enriched in CGs by KEGG pathway analysis (Supplementary Table 9). For instance, the large families of phenylpropanoid biosynthetic genes like *PAL, HCT*, *CAD*, and *Caffeic Acid O-Methyltransferase* (*COMT*) and the flavonoid biosynthetic genes like *CHS*, *DFR*, *F3’H*, and *FLS* were upregulated in all three *Les* mutants (Figure 5 and Supplementary Tables 13, 15). Besides, genes encoding laccases, which promote the oxidative coupling of monolignols to form lignin (Zhao et al., 2013), are mostly co-upregulated (Figure 5A and Supplementary Table 13). In addition, almost all genes involved in the biosynthesis of kauralexins and zealexins were upregulated in all three *Les* mutants (Figure 7 and Supplementary Table 17). Interestingly, for those benzoxazinoid biosynthetic genes, *BX1*–*BX6* are generally downregulated, while *BX10*, *BX11*, *BX12*, and *BX14* were generally upregulated (Supplementary Figure 4 and Supplementary Table 18), suggesting the differential regulation of DIMBOA-Glucoside and HDMBOA-Glucoside biosynthesis in *Les* mutants. It is possible that the upregulation of *BX11*–*BX14* may lead to the accumulation of HDMBOA-Glucoside, which was previously known to dominantly accumulate upon insect herbivory or fungal infestation (Handrick et al., 2016). Although the absence of early *BX* genes in the CGs was not expected, it represents a truly negative control supporting the relative importance of phenopropanoids and terpenoids in *Les*-conferred disease resistance. It will be also interesting to determine the mechanism of differential regulation of upstream and downstream *B*X genes in *Les* mutants in the future.

Consistent with the transcriptomic data, we detected a large amount of CMs potentially involved in plant defense response (Figure 5). For instance, significantly increased accumulation of phenylpropanoid compounds, e.g., *p*-coumaric acid, caffeic acid, and coniferyl alcohol, were detected in all *Les* mutants compared with WT (Figure 5A and Supplementary Table 14), most of those compounds have antifungal activities according to previous reports (Konig et al., 2014; Riaz et al., 2018; Yuan et al., 2019). Additionally, we also detected dramatic increases of the phenylpropanoid-based lignin accumulation in all three *les* mutants (Figure 6). It has long been proposed that lignin is involved in plant disease resistance because of its enhancement of cell wall rigidity, thus providing barriers against pathogen infection and restricting the infiltration of fungal enzymes and toxins into plant cells (Vance et al., 1980); this concept has been manifested by a recent finding that a lignin-deposited structure functions as a physical barrier similar to the Casparian strip, trapping pathogens and thereby terminating their growth (Lee et al., 2019), while MYB15 is a key TF that controls the defense-induced lignification in Arabidopsis (Chezem et al., 2017; Kim et al., 2020).

As branch products of the phenylpropanoid biosynthesis pathway, many flavonoids act as defensive compounds in response to pathogen (Middleton, 1996; Treutter, 2005, 2006). In this study, accumulation of flavonoids including dihydroquercetin, naringenin, apigenin, eriodictyol, luteolin, etc. has been dramatically increased in *Les4*, *Les10*, and *Les17* mutants (Figure 5B). Two isoflavone compounds, glycitin and pratensein, which were not reported in maize previously, were also found significantly accumulated in *Les4* and *Les10* mutant (Figure 5B and Supplementary Table 11). We were unable to directly detect kauralexins and zealexins due to technical limit, although all the biosynthetic genes appeared to be upregulated. Interestingly, the amount of DIMBOA-Glucoside was reduced in all *Les* mutants (Supplementary Figure 4 and Supplementary Table 11), consistent with the commonly downregulation of *BX1*–*BX6* genes in *Les* mutants (Supplementary Figure 4 and Supplementary Table 18). Besides, although less represented, some other potential defense-related secondary metabolites like lignans and coumarins were also detected in *Les* mutants (Supplementary Table 11). Taken together, our metabolomics study suggests that the secondary metabolites, including the phenylpropanoids, lignin, flavonoids, kauralexins, zealexins, etc., are closely associated with the enhanced disease resistance of *Les* mutants.

In this study, while the identification of SGs in each *Les* mutant gave clues to the function of each *Les* gene, the detailed analysis of the overlapped robust changes of defense-related gene expression and metabolite levels in all three *Les* mutants led to the interesting discovery of a regulatory and metabolic network associated with *Les*-related traits including spontaneous lesion formation, stunted growth, and enhanced disease resistance. Since both genetic background and timing of sampling those *Les* mutants could affect the general gene expression and metabolite changes, the regulation is not likely direct and straightforward, instead, the feedback regulation likely plays a pivotal role in amplifying the signaling, resulting in the constitutive activation of defense responses and cell death, and finally exhausting the plants. The above hypothesis is worthy of testing using some better-characterized *Les* mutant in pure background and sampling of *Les* mutants at different stages of lesion formation while introducing “days after lesion formation” as a parameter.

Based on our study, a working model is proposed (Supplementary Figure 5). Firstly, as consequences of *LES* gene mutations, a series of WRKY and MYB TFs were activated in yet unknown mechanisms. Those TFs could then regulate the expression of cell death and defense-related genes including immune receptors, defense signaling components, *PR* genes, ROS production genes, and secondary metabolite biosynthetic genes, leading to lesion formation and enhanced disease resistance. Secondly, the activation of secondary metabolite biosynthetic gene expression would result in high accumulations of metabolites including phenylpropanoids, lignin, flavonoids, kauralexins, zealexins, etc., and the metabolite change accompanied by lesion formation likely also causes amplified gene expression changes in a feedback manner. Thirdly, the stunted plant growth of *Les* mutants is probably caused by the reduced expression of photosynthetic genes followed by lower photosynthesis rate in *Les* mutants, which is also likely related to the cell death and metabolite changes. However, the proposed regulatory network is preliminary and remains to be validated and fixed in the future when the more *LES* genes are cloned and more direct evidence including the protein–protein (or DNA) interaction and genetic analysis is provided. In addition, further studies on the identified SGs and CGs will shed new light on the function of each *LES* gene as well as the general regulatory network of defense responses in maize.

## Data Availability Statement

Data of RNA-Seq in this study have been deposited in the National Center for Biotechnology Information Gene Expression Omnibus (GEO) database (accession no. GSE148220).

## Author Contributions

MG and XM designed the experiments, interpreted the data, and wrote the article. XM performed most of the experiments and analyzed the data. JL prepared the materials. LX and TF performed the pathogen test. ZD, TJ, and MC worked on the lignin measurement and RNA extraction. All authors contributed to the article and approved the submitted version.

## Conflict of Interest

The authors declare that the research was conducted in the absence of any commercial or financial relationships that could be construed as a potential conflict of interest.

## Footnotes

1 http://revigo.irb.hr/

2 http://bioinfogp.cnb.csic.es/tools/venny/index.html

3 http://suba.live/

4 http://planttfdb.cbi.pku.edu.cn/index.php

5 www.shimadzu.com.cn/

6 www.appliedbiosystems.com.cn/

7 http://csbg.cnb.csic.es/mbrole2/index.php

8 http://prgdb.org/prgdb/

9 http://bioinfo.sibs.ac.cn/plant-regulomics/

## Supplementary Material

Supplementary Figure 1 The phenotype of typical wild type, putative homozygous, and putative heterozygous plants of *Les4*, *Les10*, and *Les17*.

Supplementary Figure 2 Principal components analysis of the gene expression profiles **(A)** and metabolites **(B)** in mutant vs. wild type of *Les4*, *Les10*, and *Les17*.

Supplementary Figure 3 Distribution of metabolites measured by ultra-high performance liquid chromatography-electrospray ionization-tandem mass spectrometry (UPLC-ESI-MS/MS).

Supplementary Figure 4 Integrated transcriptomics and metabolomics analysis of benzoxazinoids biosynthesis. The changes of gene expression and metabolite accumulation in benzoxazinoids biosynthetic pathway in *Le4*, *Les10*, and *Les17* mutants compared to their respective wild type (WT). The different rows represent the different genes for each enzyme, with each column representing different genotypes and different colors indicating different gene expression levels. The number below each metabolite is the log_2_ (fold change) value. The detailed information of genes involved in benzoxazinoids biosynthesis is available in Supplementary Tables 18. BX1-14, Benzoxazinless 1-14; IGPS, Indole-3-Glycerolphosphate Synthases; IGL1, Indole-3-Glycerolphosphate Lyase 1; TSA, Tryptophan Synthase Alpha-Subunit, TSB, Tryptophan Synthase Beta-Subunit.

Supplementary Figure 5 The proposed model for the regulatory network associated with *Les*-conferred defense response. NLRs, Nucleotide Binding Leucine-Rich Repeat Proteins; PRs, Pathogenesis-Related Proteins; PRRs, Pattern Recognition Receptors; ROS, reactive oxygen species.

Supplementary Table 1 Primers used in the PCR and qRT-PCR.

Supplementary Table 2 Number of differentially expressed genes and metabolites in leaves of different maize mutant vs. wild type of *Les4*, *Les10*, and *Les17*.

Supplementary Table 3 Significantly differentially expressed genes in the comparisons of mutant vs. wild type of *Les4*, *Les10*, and *Les17*.

Supplementary Table 4 Fold change of gene expression profiles detected by qRT-PCR.

Supplementary Table 5 Gene ontology term analysis of differentially expressed genes in *Les4*, *Les 10*, and *Les17*.

Supplementary Table 6 Predicted Pathogen Receptor Genes (PRGs) in specific differentially expressed genes of *Les4*, *Les10*, and *Les17*.

Supplementary Table 7 Predicted Pathogen Receptor Genes (PRGs) in common differentially expressed genes of *Les4*, *Les10*, and *Les17*.

Supplementary Table 8 Transcriptomic comparison enrichment analysis of common differentially expressed genes.

Supplementary Table 9 Kyoto Encyclopedia of Genes and Genomes analysis of common differentially expressed genes.

Supplementary Table 10 Transcription factors in the common differentially expressed genes.

Supplementary Table 11 The detected metabolites in *Les4*, *Les10*, and *Les17* compared to their respective wild type by LC-MS.

Supplementary Table 12 Common differentially accumulated metabolites in the comparisons of mutant vs. WT in *Les4*, *Les10*, and *Les17*.

Supplementary Table 13 Fold change of gene expressions involved in phenylpropanoid and lignin synthesis.

Supplementary Table 14 Fold change of accumulated metabolites involved in phenylpropanoid and lignin synthesis.

Supplementary Table 15 Fold change of gene expressions involved in flavonoid biosynthetic genes.

Supplementary Table 16 Fold change of accumulated metabolites involved in flavonoid synthesis.

Supplementary Table 17 Fold change of expressions level for zealexins and kauralexins biosynthetic genes in *Les* mutants compared to their respective wild type.

Supplementary Table 18 Fold change of expression level for benzoxazinoid biosynthetic genes in *Les* mutants compared to their respective wild type.

Supplementary Table 19 Differentially expressed genes involved in photosynthesis in mutant vs. wildtype of *Les4*, *Les10*, and *Les17*.
