# Supplementary material for: Commonly and Specifically Activated Defense Responses in Maize Disease Lesion Mimic Mutants Revealed by Integrated Transcriptomics and Metabolomics Analysis
Source: Front Plant Sci. 2021 May 17;12:638792. doi: 10.3389/fpls.2021.638792 (PMC8165315; doi:10.3389/fpls.2021.638792)
Supplement: Supplementary Figure 1 — The phenotype of typical wild type, putative homozygous, and putative heterozygous plants of Les4, Les10, and Les17. [file Presentation_1.PPTX]

## Slide 1
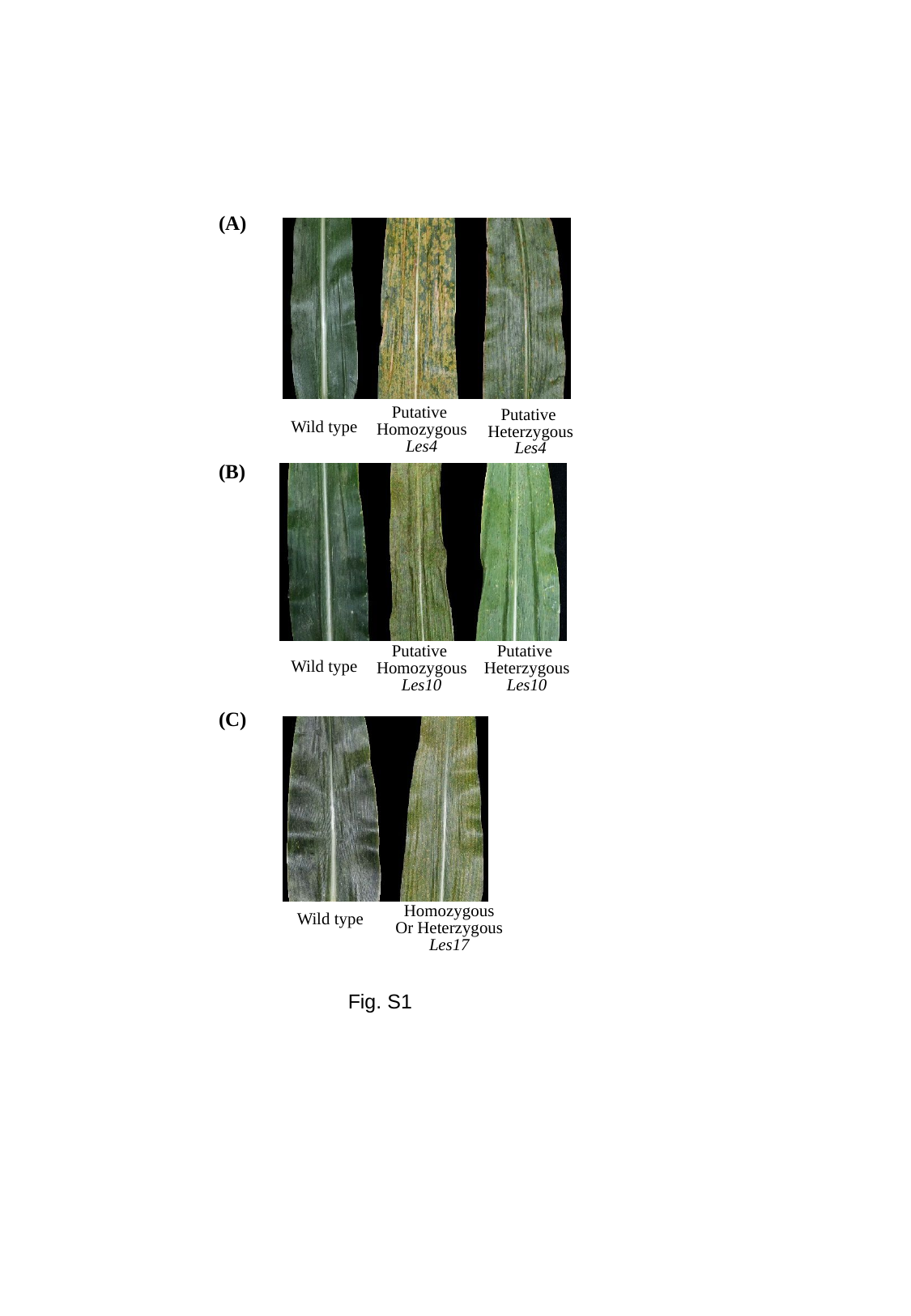

(A)
Putative
Homozygous
Les4
Putative
Heterzygous
Les4
Wild type
(B)
Putative
Heterzygous
Les10
Putative
Homozygous
Les10
Wild type
(C)
Homozygous
Or Heterzygous
Les17
Wild type
Fig. S1

## Slide 2
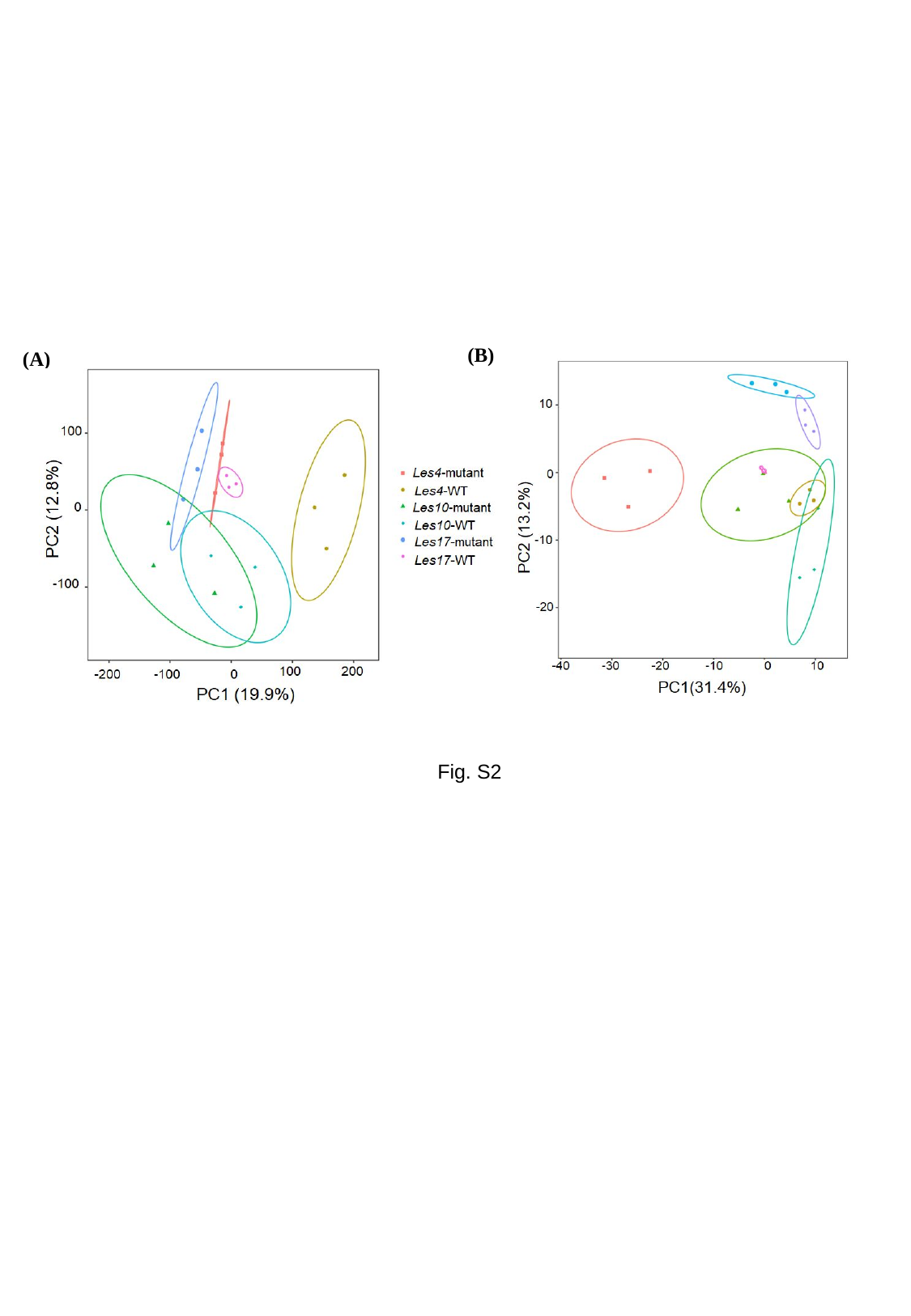

(B)
(A)
Fig. S2

## Slide 3
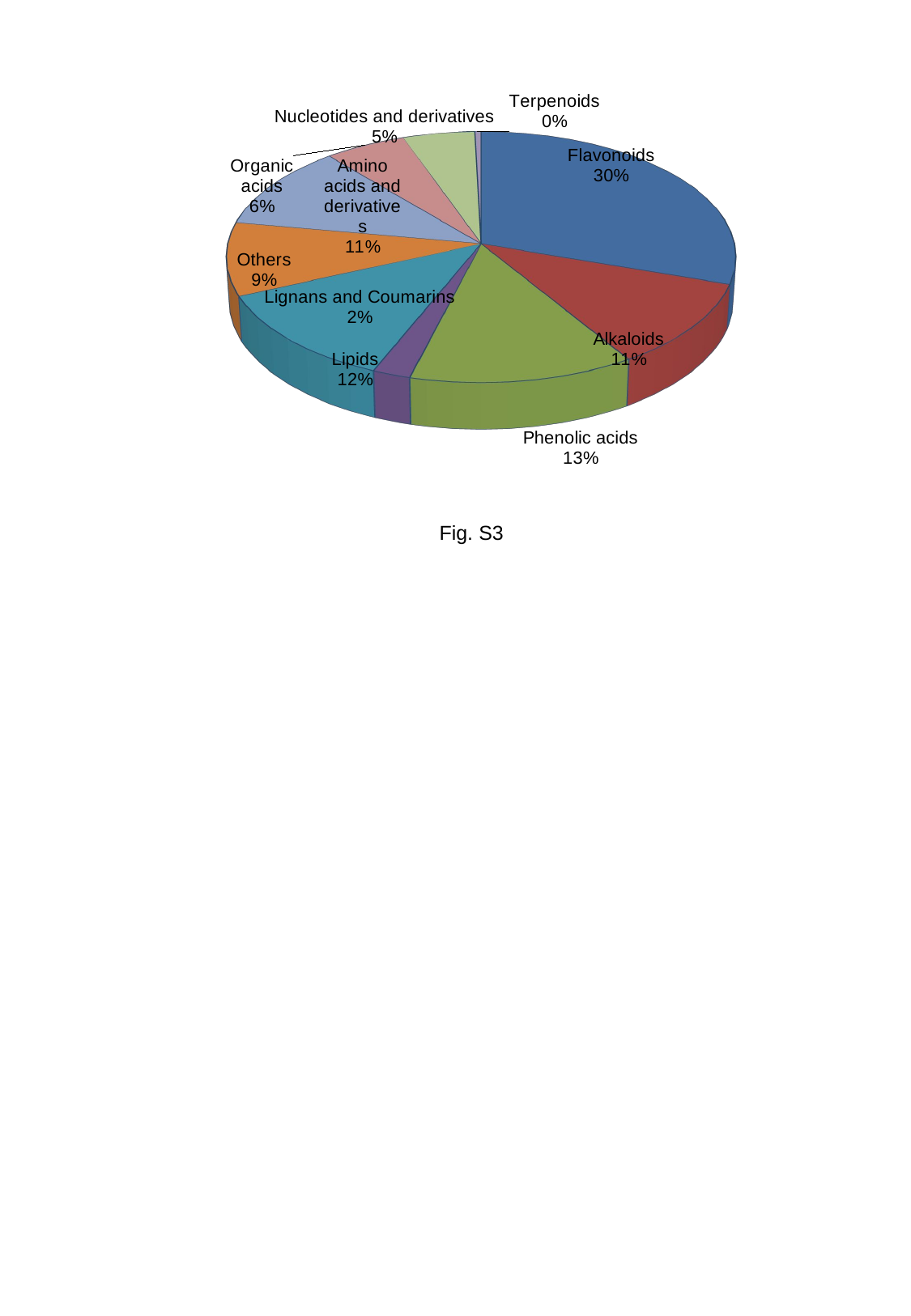

[unsupported chart]
Fig. S3

## Slide 4
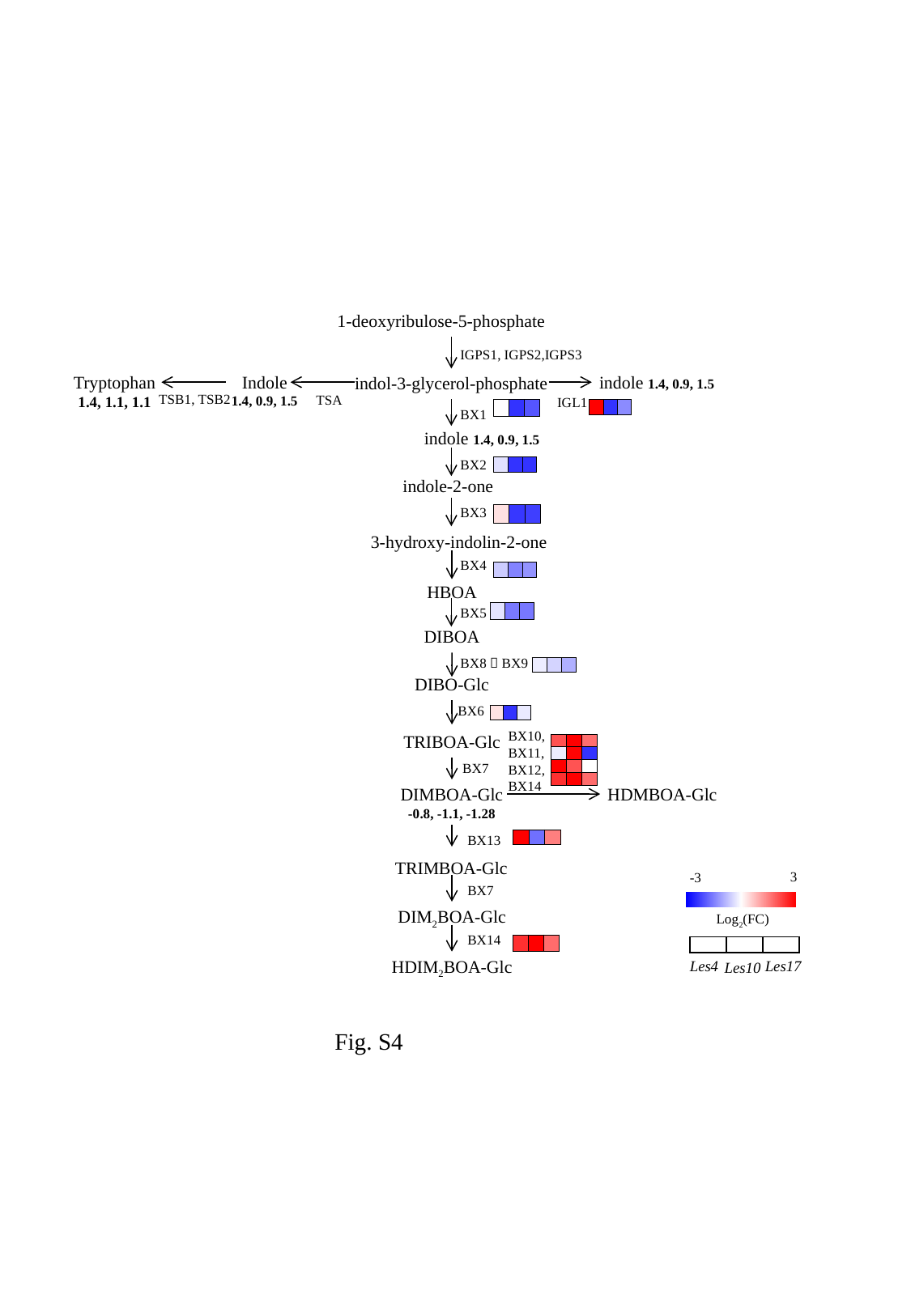

1-deoxyribulose-5-phosphate
IGPS1, IGPS2,IGPS3
Tryptophan
1.4, 1.1, 1.1
Indole
1.4, 0.9, 1.5
indole 1.4, 0.9, 1.5
indol-3-glycerol-phosphate
TSB1, TSB2
TSA
IGL1
| | | |
| --- | --- | --- |
| | | |
| --- | --- | --- |
BX1
indole 1.4, 0.9, 1.5
BX2
| | | |
| --- | --- | --- |
indole-2-one
BX3
| | | |
| --- | --- | --- |
3-hydroxy-indolin-2-one
BX4
| | | |
| --- | --- | --- |
HBOA
BX5
| | | |
| --- | --- | --- |
DIBOA
BX8，BX9
| | | |
| --- | --- | --- |
DIBO-Glc
BX6
| | | |
| --- | --- | --- |
BX10,
BX11,
BX12,
BX14
TRIBOA-Glc
| | | |
| --- | --- | --- |
| | | |
| | | |
| | | |
BX7
DIMBOA-Glc
-0.8, -1.1, -1.28
HDMBOA-Glc
BX13
| | | |
| --- | --- | --- |
TRIMBOA-Glc
3
-3
Log2(FC)
BX7
DIM2BOA-Glc
BX14
| | | |
| --- | --- | --- |
| | | |
| --- | --- | --- |
HDIM2BOA-Glc
Les4
Les17
Les10
Fig. S4

## Slide 5
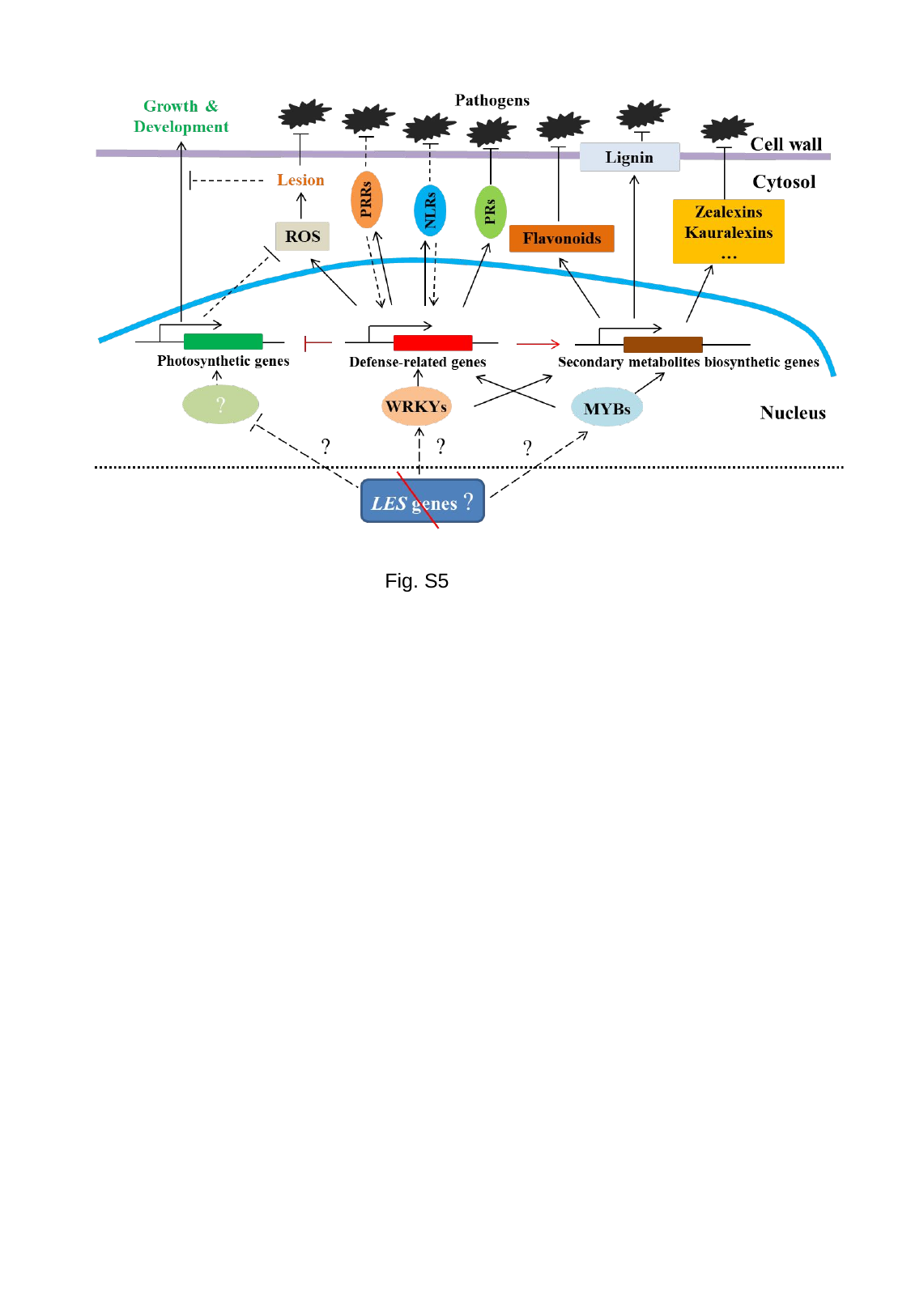

Fig. S5
